# Addendum: *Arabidopsis* ubiquitin ligase MIEL1 mediates degradation of the transcription factor MYB30 weakening plant defence

**DOI:** 10.1038/s41467-019-09341-4

**Published:** 2019-03-28

**Authors:** Daniel Marino, Solène Froidure, Joanne Canonne, Sara Ben Khaled, Mehdi Khafif, Cécile Pouzet, Alain Jauneau, Dominique Roby, Susana Rivas

**Affiliations:** 1 INRA, Laboratoire des Interactions Plantes-Microorganismes, UMR441, Castanet-Tolosan, F-31326 France; 2 CNRS, Laboratoire des Interactions Plantes-Microorganismes, UMR2594, Castanet-Tolosan, F-31326 France; 3 Fédération de Recherche 3450, Plateforme Imagerie, Pôle de Biotechnologie Végétale, Castanet-Tolosan, F-31320 France; 4 Department of Plant Biology and Ecology, University of the Basque Country, Apdo. 644, E-48080 Bilbao, Spain; 5 IKERBASQUE, Basque Foundation for Science, 48011 Bilbao, Spain; 6 Present Address: The Sainsbury Laboratory, Norwich Research Park, Norwich, NR4 7UH UK

Addendum to: *Nature Communications* 10.1038/ncomms2479, published online 12 February 2013

In Fig. 4a and Supplementary Fig. 5 of this article, incorrect portions of the same Ponceau-stained blot are displayed as loading controls in the lower panels labelled ‘Rubisco’. Updated versions of Fig. 4a and Supplementary Fig. 5 and the original Ponceau-stained membrane and corresponding western blot images are shown in Fig. 1 below.

**Fig. 1:**
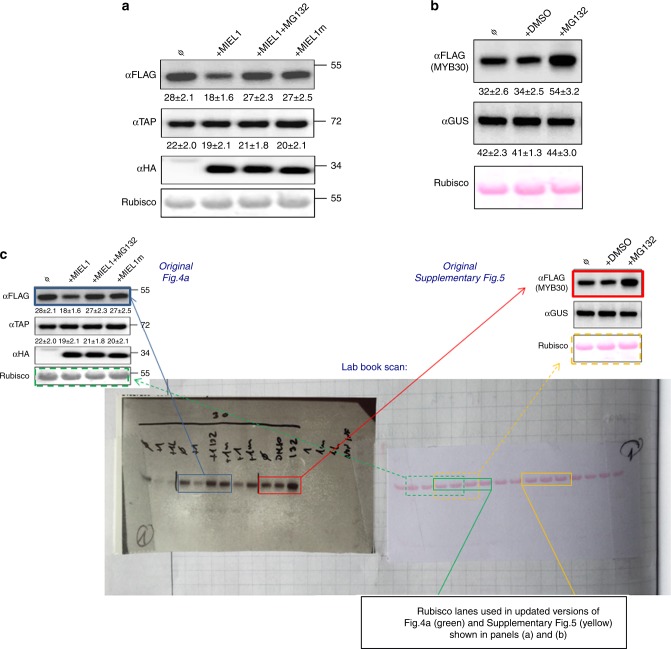
Updated version of Fig. 4a and Supplementary Fig. 5. **a** Updated version of Fig. 4a showing the correct Ponceau-stained loading controls. FLAG-tagged MYB30 or TAP-tagged MYB123 were expressed in *N. benthamiana* alone or with haemagglutinin (HA)-tagged MIEL1 or MIEL1m, and treated or not with the proteasome inhibitor MG132, as indicated. Western blot analysis shows the expression of FLAG-tagged MYB30, TAP-tagged MYB123 and HA-tagged MIEL1 proteins. Molecular mass markers in kilodaltons are indicated on the right. **b** Updated version of Supplementary Fig. 5 showing the correct Ponceau-stained loading controls. *N. benthamiana* leaves transiently expressing FLAG-tagged MYB30 or the control protein GUS were mock-treated with DMSO or treated with the proteasome inhibitor MG132, as indicated. Western blot analysis shows the expression of FLAG-tagged MYB30 or GUS proteins. **c** Original scans of Ponceau-stained membrane and corresponding anti-FLAG Western blot. The loading control panel for Fig. 4a in the original article incorrectly shows lanes 2–5 (counting from the left) rather than lanes 4–7. The loading control panel for Supplementary Fig. 5 incorrectly shows lanes 4–6 instead of 10–12

In addition, the images of yeast colonies displayed in Supplementary Fig. 2 do not correspond to the strains listed in the figure labels. An updated version of Supplementary Fig. 2 showing original images of the plates used in the yeast two-hybrid assays is shown in Fig. 2 below.

**Fig. 2:**
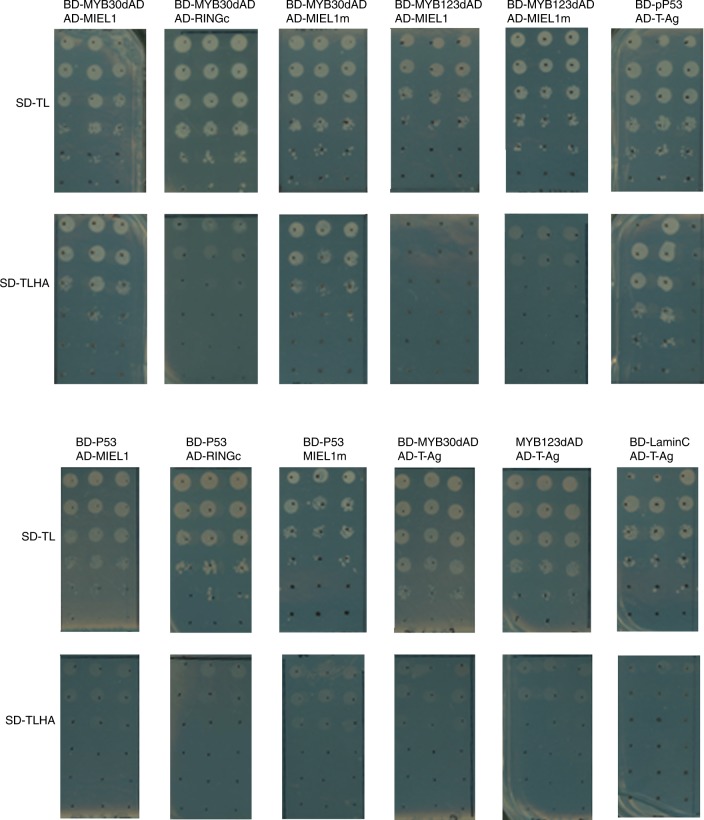
Updated version of Supplementary Fig. 2

All authors have approved this addendum with the exceptions of M.K., who could not be reached for comment, and D.R., who has declined to sign this addendum.

